# UK Public Focus Groups on Healthcare's Environmental Impacts: A Critical Analysis of Co‐Benefits Approaches

**DOI:** 10.1111/1467-9566.70058

**Published:** 2025-06-16

**Authors:** Gabrielle Samuel, Miranda MacFarlane, Sarah Briggs

**Affiliations:** ^1^ Department of Global Health and Social Medicine King's College London London UK; ^2^ Nuffield Department of Medicine University of Oxford Oxford UK; ^3^ Department of Medicine, Centre for Personalised Medicine St Anne's College and Nuffield, University of Oxford Oxford UK

**Keywords:** co‐benefit, environment, environmental harms, environmental impacts, focus groups, health, healthcare, net zero, qualitative research, UK

## Abstract

The urgency of addressing climate change has accelerated the need for healthcare to mitigate its associated environmental harms. Co‐benefits approaches are being used in policymaking to frame mitigation actions because they promise to deliver better health outcomes alongside environment benefits. Despite this, little empirical data exists on public perceptions about the acceptability and usefulness of this approach. We conducted 12 focus groups with 82 members of the UK public asking the question: what were participants' values, beliefs and experiences about the environmental harms associated with healthcare and how should these issues be conceptualised and addressed? Co‐benefits framings resonated with participants, who perceived this approach as useful for prioritising healthcare needs while valuing the environment. However, when participants tried to frame co‐benefits as a solution, they struggled to reconcile complexities. Furthermore, their discussions revealed a certain subjectivity and context‐specificity in co‐benefits framing, drawn from their own experiences and expectations of care. We emphasise paying attention to such subjectivities when developing co‐benefits policies. This could be achieved by the inclusion of public and patient voices in policymaking. Any underlying assumptions associated with co‐benefits policies—including which subjectivities are used in the framing and how tensions are resolved—must be made transparent.

## 1 Introduction

Co‐benefits is a policy framing underpinned by the idea that the positive effects of a particular policy will also produce benefits (co‐benefits) in other policy areas (Climate Change, IPPC 2014, 14). Over the past few decades, co‐benefits has emerged as a particularly useful framing to bring advocacy potential and political feasibility to addressing environmental concerns (Mayrhofer and Gupta 2016). An environment‐health co‐benefits framing has become particularly prominent in environmental policies: the promise of health benefit is viewed as an important way to gain political traction for environmental harm mitigation initiatives, especially given increasing evidence of the interrelationship between health and environment (World Health Organisation n.d.; The Academy of Medical Sciences 2021; Marshall and Allen 2023; Redvers 2021; Robinson and Breed 2019; Mayrhofer and Gupta 2016; Karlsson et al. 2020; Lemery et al. 2021; Oliveira and Thorseth 2016; Kovacic et al. 2019; Shrestha et al. 2024). Framing the benefits of environmental policies in terms of *direct* health co‐benefits is particularly appealing because of the immediate visible and tangible improvements to individuals and their communities (Holguera et al. 2020; Myers et al. 2012; Maibach et al. 2010; Workman et al. 2018; Amelung et al. 2019; Moutet et al. 2024). This includes, for example, low carbon, active transport, which improves physical health and reduces exposure to harmful pollutants; eating fewer meat products, which reduces farming emissions and is associated with lower cardiovascular and cancer risk; efficient housing, which saves energy and reduces cold and damp related illnesses; sustainable prescribing, which reduces pharmaceutical production and avoids harmful effects of over‐medicating; and protecting or enhancing biodiversity, which is related to improved mental and physical health outcomes (Haines 2017; Marshall and Allen 2023; Redvers 2021; World Health Organisation n.d.; Karlsson et al. 2020; Gao et al. 2018; Lemery et al. 2021; Shrestha et al. 2024; Marselle et al. 2021).

In the healthcare sector, a health‐environment co‐benefit framing which promises improved health benefits while simultaneously supporting the reduction of healthcare's associated environmental harms is especially important given that improving health outcomes is the primary mission of the sector. Globally healthcare reflects approximately 4%–5% total greenhouse gas (GHG) emissions, with higher income settings typically reporting greater GHG (or carbon equivalent) footprints (Karliner et al. 2020). In addition, the manufacture, production, transportation, use and disposal of medications and healthcare‐related equipment and materials (including plastics, papers, metals, hazardous and nonhazardous reagents, chemicals in building infrastructures, pesticides, food additives, electrical and digital devices and etc.) are associated with adverse effects on biodiversity, water usage, and contamination of land and water. The extent and impact of these environmental harms depends on the demand for healthcare (Talbot et al. 2024), approaches to waste management (Zikhathile et al. 2022), and practices around procurement (Davies et al. 2024), catering (Sonnino and Mcwilliam 2011) and prescribing practices and compliance (Thomas and Depledge 2015; Cussans et al. 2021), all of which contribute to the overall efficiency of healthcare systems (Andrieu et al. 2023).

Advocates are working at country levels to reduce healthcare's environmental harms, with examples including the United Kingdom's (UK's) National Health Service (NHS), the United States Practice GreenHealth^1^ and the Dutch Government's Green Deal on Sustainable Healthcare (Krens 2022). At a regional level, local hospitals are switching to renewable energy sources or generating their own renewable energy^2^, low‐emission transport to work schemes are being implemented (NHS England 2023; King’s Green Plan n.d.) and hospitals are recycling wastewater (Greenvironment 2021), reducing plastic use (Strain 2022), using low emission care pathway/device alternatives (Thiel et al. 2017; Ravindrane and Patel 2022; Alshqaqeeq et al. 2020; Ryan and Nielsen 2010; Moura‐Neto et al. 2019; Namburar et al. 2018), and creating more green space (King’s Green Plan n.d.). At COP28's health day, 143 countries endorsed a declaration promoting ‘steps to kerb emissions and reduce waste in the health sector’ (COP28 2023)^3^. The Alliance for Transformative Action on Climate includes over 80 countries pledging to strengthen the climate resilience and/or reduce the global GHG emissions of their health systems (Alliance for Transformative Action on Climate and Health n.d.). Political action is also being shaped—and is shaping—engagement amongst international agencies and advocacy groups in this area, including the World Bank (The World Bank n.d.), the Pan American Health Organisation, (n.d.) and Healthcare without Harm (Health Care without Harm 2021). The 2023 WHO Operational framework for building climate resilient and low carbon health systems provides international guidance to achieve healthcare emissions reductions (World Health Organisation 2023).

As these shifts occur, policymakers, stakeholders and scholars are increasingly promoting co‐benefits approaches that emphasise how reductions in healthcare's environmental harms can leverage societal health improvements (Bhopal et al. 2025; Rayner et al. 2025). At the same time, little research has explored public perceptions about the acceptability of such a framing. We conducted 12 focus groups with 82 members of the UK public to ask: what were participants' values, beliefs and experiences about how the environmental impacts associated with the healthcare system are and should be conceptualised and addressed? Participants spontaneously discussed co‐benefits approaches as a way to address the healthcare sectors' environmental harms because they viewed co‐benefits as allowing the prioritisation of patients' health needs while also promising to have environmental benefit. This was particularly appealing compared to other approaches, such as trade‐offs, that required the moral balancing of the incommensurable values of environment and health. However, as participants explored the co‐benefits framing in more detail, complexities emerged, including issues associated with the lack of clarity, subjectiveness and context specificity of the term, as well as with the ongoing requirement to make value choices at certain levels. We argue that these findings offer important reflections for developing co‐benefits policy approaches, and we discuss the implications of this.

Before we describe and discuss our findings, we provide a review of the literature around co‐benefits and introduce the policy context of the UK healthcare sector as it applies to addressing its environmental harms.

### 1.1 Co‐Benefits Approaches

Co‐benefits initially emerged in the 1990s as a way to align environmental and economic goals but has since expanded across fields and sectors to offer policy options to align environmental priorities to other goals, by delivering mutually beneficial outcomes, resolving tensions, and addressing issues of complexity (Mayrhofer and Gupta 2016). The accelerating use of this concept has made it a central tenet of environmental policy strategies at both the national and international levels. Given its increasing policy prominence, a lively body of academic scholarship has emerged around the co‐benefits concept. Most of this work centres on two challenges: first, how to measure and assess co‐benefit relationships and policy measures—including issues with how best to model data, which variables to use in analysis and how to account for unintended consequences—and second, issues around implementing co‐benefits approaches in practice (Moutet et al. 2024; Mayrhofer and Gupta 2016; Oliveira and Thorseth 2016; Robinson and Breed 2019; Redvers et al. 2024; Dinh et al. 2024). In terms of the latter, concerns have been voiced about systematic and organisational barriers associated with a lack of resources and capacity (Workman et al. 2018; Robinson and Breed 2019; Redvers et al. 2024; Samuel 2023), siloed thinking, a lack of integration between departments working on different policy objectives and the use of different technical language and political realities across these departments (Workman et al. 2018; Karlsson et al. 2020; Redvers et al. 2024; The Academy of Medical Sciences 2021).

Alongside this scholarship, a sociological critique of the co‐benefits approach is also emerging. Mayrhofer and Gupta (2016) have tracked the evolution of the term, arguing that its adoption, which has far outpaced other terms, was accelerated by its positive framing, where ‘benefits’ suggests gains rather than losses and cuts. Oliveira and Thorseth (2016) have argued that ‘co‐benefits consistently states facts and numbers that have created a shared belief, especially present in policy documents, that co‐benefits are good and desirable….the idea of co‐benefits itself holds a latent notion of some kind of rightness’. Kovacic et al. (2019) analysed this in detail through their exploration of circular economies—a co‐benefits approach which promises to overcome the gap between environmental problems and economic opportunities. These authors argue that circular economies remain appealing not because of their content—which often remains ambiguous and varies between policy objectives—but because of the way they contribute to socio‐technical imaginaries that frame them as easy‐fix solutions to complex problems. This solutionism framing (rather than a framing of an imperfect *process*), they say, leaves little space for critical thinking around the concept (p176), nor the fact that such an approach needs to consider multiple priorities, outcomes and potential barriers with all the associated complexity (Robinson and Breed 2019; Mayrhofer and Gupta 2016; Karlsson et al. 2020). Aligning with scholars who have explored co‐benefits more generally (Karlsson et al. 2020; Oliveira and Thorseth 2016; Mayrhofer and Gupta 2016), these authors argue that this imaginary perpetuates because it allows co‐benefits to ‘be anything’ (Kovacic et al. 2019, 75), subject to the values and experiences of those constructing the (policy) problem (Bacchi 2009). Legitimate concerns such as for whom, when, and where co‐benefits are realised are given little attention. They stress that contested knowledge and scientific uncertainty accompanies decision‐making, and that imaginaries hide any negative implications of co‐benefit interventions, including unintended negative effects and/or uncertain outcomes (Kovacic et al. 2019, 176).

Although this scholarship emphasises the heterogeneity and sometimes vacuous use of the co‐benefits term in a way that often ignores context and specificity, other sociological and ethical scholarship has attempted to critically engage with the notion of co‐benefits in other ways. Oliveira and Thorseth (2016) argue that policymakers' different understandings, definitions, and interpretations of co‐benefits leave questions about who decides what a co‐benefit is and how it gains ethical significance. These authors explain that while some benefits may be attributable to rights (e.g., health needs), others may be less tangible (aspects of well‐being, preferences and desirable goals) and therefore need to be morally analysed to understand the possibility of plausible justification. Other scholars are concerned that co‐benefit approaches could be used to ‘sell’ particular policies in an opportunistic way (Dubash et al. 2013), can obscure complexities of decisions that will still need to be made (Mayrhofer and Gupta 2016) and in reality, rarely achieve win–wins and/or come with other costs. In the context of healthcare, Redvers et al. (2024) describe how, while telemedicine is advocated as, on the one hand, reducing emissions associated with travel, and on the other hand, supporting improvements in patient experience (e.g., faster and more convenient access to care), at least some studies have suggested that such approaches may lead to overprescribing and/or longer times to discharge, with a preference in some instances for face‐to‐face appointments (Garrett et al. 2024; Vestesson et al. 2023; Samuel et al. 2024). Furthermore, concerns have been raised that positive effects can mask unfairness, leading to equality issues (Shrestha et al. 2024; Moutet et al. 2024), as well as exporting negative impacts to other countries (Oliveira and Thorseth 2016; The Academy of Medical Sciences 2021).

Finally, scholars point to co‐benefit approaches being bound up with economic rationalities—that they are symptomatic approaches which fail to attend the root cause of climate change and other environmental harms (i.e., development). Therefore, although such policies may lead to incremental improvements, if the prevailing mindset is one of ever‐increasing consumption, environmental concerns will remain unaddressed (also known as the rebound effect) (Widdicks et al. 2023). In fact, Workman et al. (2018) argue that the policymaking process is regularly ‘ruled by dominant narratives, economic and political structures, or by the interests of the most powerful players’ ((Cáceres et al. 2016) cited in (Workman et al. 2018)) and that environmental concerns will not be considered until impacts become immediate and visible.

Nevertheless, co‐benefits is increasingly being used as a framing in the healthcare sector, to which we turn to below.

### 1.2 Co‐Benefits and the UK NHS

Globally, the NHS has traditionally led the way in addressing its environmental harms. In the UK, each devolved NHS has net zero targets, albeit with approaches varying between nations^4^. These commitments are legally backed by the UK's Climate Change Act 2008^5^ and underscored by subsequent healthcare specific legislation within each devolved nation. For example, in England, the Health and Social Care Act^6^ obliges the promotion of an environmentally sustainable healthcare system. NHS Trusts and Integrated Care Boards (ICBs) are required to have their own policies, described as a ‘Green Plan’ by Greener NHS.^7^ In Scotland, the Climate Change (Scotland) Act 2009^8^ and the Climate Change (Emissions Reductions Targets) Act 2019^9^ and 2024^10^ produce obligations for NHS Scotland, as a public body, to lower its emissions. The most recent NHS Scotland Climate Emergency and Sustainability strategy (20.2–26) must be delivered by Health Boards and Integrated Joint Boards in partnership with Community Planning and Regional and Local Resilience Partnerships (NHS Scotland 2022). The Wellbeing of Future Generations (Wales) Act 2015^11^ and the Environment (Wales) Act 2016^12^ obliges public bodies, including NHS Wales, to consider enacting net zero commitments and maintain or enhance biodiversity and ecosystem resilience in policy and practice decisions, some of which are outlined in the NHS Wales Decarbonisation Strategic Delivery Plan (20.1–2030) (Carbon Trust 2021). Northern Ireland NHS does not currently have a decarbonisation strategy, though it falls under the Climate Change Act (Northern Ireland) 2022.

In practice, since 1990, NHS England has reduced its healthcare associated emissions by 26% (Tennison et al. 2021). Initiatives have included reducing emissions associated with buildings, estates and facilities; medical infrastructure; travel; and electronic devices such as freezers, lights and computers (NHS England 2018). Beyond emissions, the total water footprint of the UK NHS has been reduced by 21% from 2010 (NHS England 2018). At the same time, health and social care is declining in the UK, characterised by growing stresses across services (Ham 2023). In parallel, UK studies have shown an overall decline in public support for the NHS prioritising environmental considerations between 2021 and 2023, with members of the public more concerned about the NHS addressing staff workload (40%), increasing staff numbers (39%), and improving waiting times for routine services (34%) (Callan et al. 2023). The public are concerned that addressing environmental issues would lead to ‘more top–down regulation, restrictions in personal consumption, and lower quality products and services’ within the NHS (National Institute for Health and Care Excellence (NICE) 2023). Taken together these perspectives might partially explain why the co‐benefits approach is appealing in the UK and has been used as a framing to address environmental concerns in the NHS. For example, the English NHS Net Zero delivery plan (NHS 2020) makes various references to the ‘significant health benefits’ to patients and staff which may be secured by advancing the net zero agenda within healthcare. NHS Scotland's Climate Emergency and Sustainability Strategy 2022 (NHS Scotland 2022) explicitly refers to co‐benefits as part of adaptive actions, sustainable care pathways and efforts to evaluate policy decisions. Although neither the Wellbeing of Future Generations Act (2015)^13^ nor the NHS Wales Decarbonisation and Sustainability Plan (20.1–30) (Public Health Wales 2024) use the term ‘co‐benefit’, both implicitly reflect this approach through commitments to sustainable development, advocating for low carbon solutions to social challenges which simultaneously seek to improve poverty, health inequality, and mental and physical health. Finally, The Health Foundation in the UK emphasises that effective climate change and environmental harm mitigation strategies within the NHS requires a systematic approach and better coordination of policy levers to ‘maximise’ co‐benefits (Callan et al. 2024); scholars are also increasingly stressing the importance of co‐benefits approaches (Rayner et al. 2025). Therefore, it is considered that in the current socio‐political context, co‐benefits may help future‐proof the NHS (Issa et al. 2024) and offer a means to legitimise efforts to decarbonise healthcare, even while the NHS faces other persistent challenges (Burt and Cameron 2024).

## 2 Methods

### 2.1 Recruitment

Twelve focus groups were held online (*n* = 4) and in person (*n* = 8) across the UK, with the aim of including individuals with varied demographics (see Table 1). The locations of in‐person focus groups were South London, North London, South Leeds, North Leeds, Norwich, Swansea, Glasgow and Edinburgh. All were hosted in public venues and/or community centres (e.g., libraries, community centres and arts spaces) to ensure visibility within the local community and lessen barriers to participation amongst people who might not otherwise have attended outside of familiar and trusted settings.

**TABLE 1:** Demographic information associated with participants.

Demographic	Number of participants	Total self‐reported^a^
Gender		
Female	54	82
Male	27	
Non‐binary	1	
Age		
18–25	9	74
26–35	14	
36–50	18	
51–65	21	
66–75	9	
76 and over	3	
Ethnicity^b^		
White British	34	69
White other	16	
Asian	10	
African	3	
Black British	2	
Scottish	2	
British	1	
Middle Eastern	1	
Country of residence		
England	53	82
Scotland	19	
Wales	5	
Northern Ireland	5	
Occupation of main household earner when aged 14^b^		
Professional	35	71
Working class	24	
Intermediate	9	
Other	2	
Prefer not to say	1	
Eligible for free school meals		
No	33	68
Yes	14	
Not applicable (attended school outside the UK)	17	
Finished school pre‐1980	3	
Unsure	1	
Type of school attended between ages 11–16		
State run or state funded school	53	72
Attended school outside the UK	10	
Independent or fee‐paying school	5	
Independent or fee‐paying school	2	
State funded & private	1	
Mixture of schooling abroad and state run school	1	
Employment status		
Employed full‐time or part‐time	32	72
Self‐employed or freelance	8	
Unemployed	6	
Family carer	1	
On sickness benefit	1	
Retired	17	
Student or training	5	
Disabled	1	
Other	1	
Highest level of education attained		
Masters degree/PhD	25	68
Bachelors Degree/HND	23	
Certificate of higher education	2	
A level or similar (e.g., apprenticeship)	9	
GCSE (or O Level/CSE)	8	
No formal qualifications	1	

^a^ Because providing demographic information beyond country of residence was optional at the point of registration, total self‐reported figures are sometimes lower than the total 82 people who took part in focus groups.

^b^ Self‐reported data was collected using free‐text and has been grouped into categories to reflect the information provided.

Participants were recruited through online and print advertisements in local newspapers, social media groups, libraries, community groups, and university networks. Local institutions, voluntary networks, and action groups local to each host venue were also contacted to help promote the opportunity through community newsletters, notice boards and social media channels, some of which were at the national level (e.g., Patients Association Newsletter). Finally, we commissioned *Roots Research* to support recruitment in Leeds, Norwich, Edinburgh, and online groups (and, in particular, to reach participants in Northern Ireland). Their service permitted purposive selection of participants, ensuring that a range of demographics were included in each focus group. Across all focus groups, Roots recruited 21 out of the total 82 participants (with three no shows). All participants were offered £50, plus travel expenses (up to £10).

Our recruitment process used an online registration page, which included information about the project and a short form where participants could express their interest, as well as optionally provide additional demographic criteria. This included age, gender, ethnicity, employment status, disability, and socio‐economic background. We included QR codes on print materials which participants could scan to reach the online registration page and form. Potential participants were then emailed information about the project, including a participant information sheet and consent form to sign. Those participants who chose to take part in a focus group were emailed a one‐page information sheet about the topic, along with an optional pre‐workshop activity which helped them consider the various steps involved in a healthcare encounter—experienced by themselves, a friend or family member—by mapping or visualising the experience and annotating it with where they thought environmental impacts (positive and/or negative) were relevant.

Focus groups were moderated by two researchers, were 2 hours in duration, with a 5‐min coffee break, and were video and audio recorded (online and in person). Recordings were transcribed based on words (rather than body language and/or gaps in speaking or intonations), though aspects of agreement and disagreement were also paid attention to. Video recordings were deleted once transcription had been completed.

### 2.2 Focus Group Schedule

The focus group schedule comprised three exercises: a mapping exercise, a speculative headlines exercise and an imaginary exercise. The mapping exercise was loosely based on published qualitative research (Lupton and Michael 2017). Participants were asked to name (and implicitly to gender) an imagined 35‐year‐old patient who was presenting with a persistent cough, and describe all the steps they perceived would be involved in making and attending a primary care appointment, including any follow up tests and/or hospital appointments. One of the researchers visualised the steps on a board (physical or Miro). Once drawn, participants were asked to indicate potential positive and negative environmental impacts of each of the steps, with discussions particularly attending to participants' experiences, and perceptions and awareness of environmental concerns in the context of healthcare. The second exercise presented fictional news headlines of healthcare and the environment and asked participants for immediate reflections and perspectives. Headlines were kindly gifted by the Health Foundation‐Liminal Space collaborative project ‘Net Zero NHS: Imagining the future’,^14^ with slight modifications to adapt to our research aims. The final exercise asked participants to imagine a future version of the healthcare system that contained all values important to them—including and beyond those that had been discussed—and to reflect on what this future healthcare system would be like (and whether/how it differs to current healthcare).

### 2.3 Analysis

Analysis was based on a collaborative methodology published by the Solpan consortium (Zimmermann et al. 2022). Focus group moderators independently documented initial reflections and perspectives of each focus group in a memo. Researchers not in attendance listened to the focus group and developed memos from listening after the event. Memo reflections were discussed in a 2‐h online meeting of all three researchers. From this, a deductive coding structure (two hierarchical levels) was developed for thematic analysis. Analysis was conducted in NVivo. Two researchers independently trialed this structure on a transcript and all three researchers discussed the findings and made modifications to the coding schedule during an online meeting. The coding schedule was then applied to another transcript by one researcher. Further modifications were applied to the coding schedule during another online meeting. This coding schedule then became the master coding schedule. Transcripts were divided between the three researchers to code using the master schedule. During coding, inductive new codes were added and discussed at weekly meetings to alert other researchers to their additions. Once a transcript was coded, a second researcher who had not conducted the initial analysis checked the initial coding, and discrepancies were discussed in online meetings, where relevant—though there were no major discrepancies. No differences were noted between demographic criteria.

### 2.4 Limitations

Recruitment sought to capture people with varying levels of engagement and/or interest in environmental sustainability and/or climate change. Self‐selection bias is a factor which should be considered in the context of this project. Mitigating steps included emphasising—through venue staff—that participation did not depend on pre‐existing knowledge or interest in environmental issues relating to healthcare. Participants were also offered an incentive, though we note that this approach has its own limitations, including biased enrolment (skewed towards participants within lower socio‐economic groups for whom the incentive is more significant (Resnik 2015). However, overall, our participants did appear to overrepresent those with an interest in environmental issues. Furthermore, although our cohort reflects diversity across demographics, most participants were white and educated to degree level or above. Finally, this study was UK‐based and its findings cannot be generalised to publics elsewhere.

### 2.5 Ethics Approval

Approval was granted by King's College Research Ethics Committee (LRS/DP‐23.24–39889).

### 2.6 Findings

Although most participants had not previously considered UK healthcare's implicated environmental harms, they concurred between them that this was an important issue to address. They thought about different approaches to providing plausible and/or acceptable solutions, such as trade‐offs and consequentialist arguments. However, participants were concerned that these approaches often placed health and environmental values in tension with each other, requiring difficult choices to be made—choices in which they felt they would always prioritise health needs. To avoid these tensions, participants shifted to discussing an environment‐health co‐benefits approach, often in the context of three recurring areas: reducing avoidable waste, preventing illness and holistic care. However, as participant discussions progressed, this framing's utility seemed to be context specific because participants subjectively defined co‐benefits in terms that reflected their own experiences and expectations of care. In the following sections, we foreground issues of inconsistency, ambiguity and subjectivity inherent to the co‐benefits framing, before finishing with a final section exploring the importance of questions associated with *who* is defining co‐benefit strategies.

### 2.7 Avoidable Waste

Participants felt that reducing avoidable waste in the healthcare system could decrease environmental harms, while simultaneously improving efficiency, saving time and delivering financial gains. They had observed material waste which was perceived as avoidable or unnecessary both within and outside clinical encounters. Participants spoke about wasted paper and packaging, insufficient pathways for medication recycling, and ephemeral use of disposable single use plastics (PPE, tourniquets, needles, sample bottles and other medical equipment): ‘fresh plastic gloves put on and then discarded even though they haven't actually touched a patient … it's just crazy’ (female, participant C, group 2). They also highlighted wasted energy and water usage in buildings. In fact, the temperature of buildings was perceived as a big energy cost that they felt, if addressed, could have the co‐benefit of improving conditions for patients and healthcare staff as well as decreasing environmental harms.

Participants also characterised care pathways as a focal point for avoidable waste. They provided examples of poorly coordinated visits for multiple (sometimes unnecessary) appointments and unnecessary visits to healthcare services; both of which, if addressed were perceived to be able to improve health outcomes as well as reduce environmental harms. One participant in Group 5 described how limited resource availability and capacity at her local surgery resulted in an additional appointment—and journey—to the hospital, just to access, in her words, ‘a ruler’:

I once had a thing on my hands…so I went to my doctors….and they have to measure it, but they didn’t have a ruler in the building……So they made me an appointment for the main hospital….And that’s to go all the way up there, and you think all the resources of going to a hospital….just because they didn't have a ruler at the surgery….

(female, participant C)

At the same time, participants' definitions of ‘avoidable’ and ‘unnecessary’ were subjective—dependent on the vantage point held within, or outside, the healthcare system. In one example, focus group 4 participants reflected on their perceptions of unnecessary versus necessary medication for depression. Although one male participant in Group 4 (participant C) felt that managing depression by ‘popping pills’ (rather than exercise approaches) was unnecessary, another participant in this group—who had experienced depression—emphasised the importance of medication, especially when ‘really, really depressed [so that you] can't get out of bed’ (male, participant F). In another example, a participant in group 2 considered the letters they received from the healthcare service to be a form of unnecessary waste because she already received digital communications which she felt offered a more appropriate and less environmentally damaging form of communication. In response another participant shared an alternative view from a healthcare professional perspective, which pointed to the importance of repetitive reminders mitigating the chance of patients missing appointments—something that was perceived to equate to a far greater waste of resources:

from the other side [as a HC professional] … you do find … a lot of duplication is placed into the system, for example, a letter plus a text message, because of the ‘did not attends’… [But] then if you’re talking about both financial cost and carbon footprint of a, of an individual who fails to attend, far exceeds the cost of sending a letter and a text.

(female, participant D)

Therefore, although participants considered addressing the issue of avoidable waste as having co‐benefits for energy reduction and improvements in efficiencies, healthcare outcomes, and cost savings, the way in which these benefits were framed was an inherently subjective endeavour.

### 2.8 Preventing Illness

Participants repeatedly emphasised how reducing the burden of disease across the population could act as a co‐benefit for reducing environmental harms because improving health would, in their view, lead to less—or less intensive—use of the healthcare system in the long‐run:

we’re going very much down the healthcare route of treatment, could we maybe rewind back to prevention as well. So, healthcare instead of focussing on hospital building, treatment, doctors, could it look into how we’re going to help the environment through possibly reducing the people that need this level of sick care…if we’re saying that we’re polluting through the healthcare system okay, how do we try and minimise that before we even get to that.

(male, participant B, group 3)

Participants envisaged a range of ways this healthcare burden could be reduced. For example, early diagnosis through screening would prevent the development of serious ill‐health and reduce the need for more energy and resource intensive treatment: ‘if someone, you know, whatever ails them is diagnosed and sorted quickly, it would actually save a lot of time, energy, waste in the future…’ (female, participant C, group 12). Participants also described reducing healthcare burdens by avoiding disease through addressing social determinants of health. A female participant from group 8 explained, ‘[not] just sort of putting in like the anti‐smoking, but stuff about food and food production, to make sure that our population is eating healthily’ (participant E). This same participant, and others, also spoke about reducing the burden of disease by nudging individuals to make choices that promoted their own health, framing this burden in terms of individual responsibility that needed to be addressed through ‘self‐management’: ‘it's sort of taking it back to basics…in terms of health and self‐management’ (female, participant E, group 8).

At the same time, participants lacked confidence in articulating the characteristics of what such preventative measures might look like in practice. There was a recognition that many preventative measures are associated with ‘causes of causes’ (Rose 2008; Phelan et al. 2010) and that the purview of addressing these would extend far beyond the NHS or even public health, though they were unsure who exactly would be responsible. A male participant in group 3 described the NHS as just one part of a larger mechanism for designing and delivering a preventative strategy, which could operate at individual (‘going to the gym’), sector wide (‘schools’), national or international scales (‘our entire food pyramid system’):

I think they [the NHS] can be a facet within [the responsibilities to reduce healthcare burden] …. I think it does come from higher up as well. It'll need funding, it will need initiatives being put in place. It will need knowledgeable people giving information about what they think is going to be the preventative measure. Because does it mean going to the gym or does it mean we change our entire food pyramid system? Does it mean that we teach in schools? Does it mean….

(participant B)

Furthermore, participants' co‐benefits framing sometimes broke down, requiring a difficult balancing of environmental and health values. As tensions emerged, participants reflected on the need to weigh up these different values. Focus group 5 participants discussed how preventative measures through the use of face masks could decrease infectious disease transmission, lowering infection rates and therefore healthcare service burden. At the same time, new tensions emerged between the value of using masks to reduce infection rates and increasing material waste (due to disposal of used masks) from adopting this approach:

Participant G (male): One positive—I thought of, is that if the guy’s got a cough, if it was infectious—it could be tuberculosis for example, by going to the GP appointment, and getting the appointment, and getting the tests done, it might well save other people being infected. So that’s positive [so there will be less burden on the health system].

Participant A (male): Yeah, the masks are obviously not good for the environment, but they are in a way that it’s not spreading diseases.

Participant G (male): That’s right … it’s a balance, isn’t it?

In this example, although a ‘win–win’ scenario is described (on the one hand, preventing infection; on the other hand, reducing the healthcare burden and therefore associated environmental harms) and appears to simplify value trade‐offs, it does not resolve them. The persistence of such tensions—between present and/or future health outcomes and environmental harms—is something participants' discussions often returned to.

### 2.9 Holistic Approaches to Care

Participants viewed holistic care as having the co‐benefit of improving health outcomes while reducing healthcare's environmental harms. This was because holistic care was described as clinical encounters in which doctors listen to patients and recognise that they are unique human beings who live in specific socio‐political contexts with particular personal and emotional needs. By listening to patients and addressing their specific needs, participants suggested that doctors would be able to prescribe the most appropriate medical/nonmedical treatment (leading to better health outcomes), and limit unnecessary waste from interventions that do not improve health:

if they just spent a little more time and did a little bit more, you would save a lot of time, resources, etc. Because they would find the problem.

(female, participant E, group 10)

Although participants were (nearly) united that holistic care meant recognising their needs, the term was often subjective and became a catch‐all solution for each participants' own dissatisfaction with healthcare service experiences: for some, receiving a quick diagnosis was holistic care, for others, a more accurate diagnosis after comprehensive tests was considered holistic. Yet, others, again, viewed holistic care more in terms of social prescribing and/or including complimentary medicine. These perspectives then shaped how participants framed co‐benefits. For example, when participants reflected on their own experiences of receiving diagnostic test results and/or prescriptions that led to a diagnosis or alleviated symptoms, they were considered a necessary use of resources, and part of holistic care. When tests failed to produce answers, or prescriptions proved ineffective (or unnecessary), this was attributed to a lack of joined up thinking/information sharing and not ‘holistic’. In the extracts below, a female participant from group 10 (participant E) described the ‘wasteful process’ that they had been through, having investigative tests—including an x‐ray and blood tests—which ultimately excluded differential diagnoses. In contrast, participant A, a female in group 6, described a similar battery of testing and triage to diagnose a problem with her leg. Although acknowledging the waste incurred as part of the process, this was viewed as appropriate patient care because it helped provide a diagnosis, which was later treated:

I was told that I have to have a chest X‐ray….and then they decided to take blood, because they thought it might be something…they came up with a conclusion, and said, I didn’t have things…. So it’s kind of, it’s a wasteful process. Because really, if they just spent a little more time and did a little bit more, you would save a lot of time, resources, etc. Because they would find the problem….

(participant E, group 10)

I was triaged and blood tested…they had to dispose of these things that they used….I had an appointment the next day… for further tests…and all that sort of waste stuff. And an ultrasound scan of the leg… you get this long paper towel they put out…I’m kinda glad they disposed of it, and I didn’t have the one that the person before…Then I was advised…to go to the pharmacy [post diagnosis] and I’d get 24 single use injections… and I went away happy.

(participant A, group 6)

Thus, the way in which participants viewed holistic care as a co‐benefit for reducing environmental harms associated with healthcare (through being able to ensure the best treatment possible first time around, therefore avoiding unnecessary waste) was dependent on their understanding of what holistic care meant in practice. Their understanding was shaped by their healthcare experiences, expectations and/or preferences about their care.

### 2.10 Importance of Who Is Framing the Co‐Benefit Approach

Participants' discussions suggested that a co‐benefits framing required consideration of *who* was proposing such a framing. This was particularly prominent in participants' reflections on speculative headlines that were presented to them in one of their focus group activities. In one example, participants discussed the following headline which promoted a co‐benefits approach: ‘The NHS will today shut four hospitals…a move that promises reductions in carbon emissions as well as better access to care [with the aim of] moving services into local community health’. In response to this headline, and when presented with other fictitious headlines detailing government policies aiming to mitigate the environmental harms associated with the healthcare system, participants expressed a range of scepticism and mistrust towards the government, and sometimes towards the NHS. They worried that policies had been designed with a co‐benefits approach to obscure ulterior motives, reflecting economic priorities rather than public or patients' interests. Some participants described such policies as opportunistic—designed to cost‐save or advance resource limiting measures which would otherwise be unpopular with members of the public. In these instances, the overarching perception was that health was not the first priority, and that environmental concerns were positioned to deflect from the intended cost cutting purpose of interventions (‘I'm sceptical too! Four hospitals closing just sounds like cost‐cutting and green issues are just an excuse’ (male, participant A, group 10)):

Just the first thing for me is I’m just suspicious, is it about the better access to care—for whom? Why is it better? How is it better? Maybe it might be better environmentally, if it’s closer to you, you might have more options to get there in an environmentally friendly fashion. Yeah. But, you know, what's not in here is for cost reductions. And I can imagine that would be behind this kind of initiative.

(female, participant A, group 6)

As such, notions of trust were crucial in participants' reflections on the framing of co‐benefits framings.

## 3 Discussion

Most participants had not previously considered the environmental harms associated with healthcare, though when prompted to think about them, participants readily engaged with the idea of co‐benefits, and saw it as a valuable framing for addressing environmental issues while keeping the need for health benefits in mind. In fact, they asserted that because health should always be prioritised, co‐benefits approaches allowed an uncompromising focus on patient health while also permitting a mutually beneficial promotion of an environmental agenda. In this way, our findings provide evidence that the co‐benefits concept, which has been developed and promoted within policymaking and academic discourse (Redvers 2021; Robinson and Breed 2019; Royal College of Physicians 2024; Mayrhofer and Gupta 2016), can resonate with members of the public.

At the same time, our findings illustrated the subjective nature of any such co‐benefits framing, and in particular how participants' contextually specific interpretations of meeting healthcare needs affected their beliefs on how to mitigate healthcare's environmental harms through co‐benefits approaches. This, in turn, was shaped by their healthcare experiences and expectations. This was best reflected in the idea of holistic care. Reflecting similar observations of the term's use by other scholars (Bullington and Fagerberg 2013), participants used the concept as a catch‐all term under a wide (and nebulous) definition. This meant that when attempting to advance a co‐benefits approach using this concept, participants had different beliefs about which healthcare interventions should be considered holistic, dependent on their own perceived care needs.

The importance for policymakers to pay attention to the subjectiveness inherent in co‐benefits framings—as highlighted by our participants—has been epitomised in the recent political discourse around the Green party's health policy on caesarean sections (Cosslett 2024)^15^. The policy framed changes to maternity services in terms of addressing the co‐harms (Haines 2017; Lemery et al. 2021) of ‘expensive and, when not medically required, risky’ caesarean sections. Criticism, however, focused on the policy's apparent ideological—rather than evidenced—basis, and rested on the definition of the terms ‘medically required’, ‘medically unnecessary’ or ‘clinically irrelevant’. This terminology excludes consideration of patients' experiences and values, and the backlash that followed illustrates differences between patient and clinician perceptions of risk (Montgomery and Fahey 2001), quality of care (Gourevitch et al. 2017), and the influence of previous experiences and socio‐cultural norms on women's decision making (Coxon et al. 2017). Furthermore, although the major discourse around the policy did not focus on environmental considerations, writing in the Guardian UK newspaper, Rhiannon Lucy Closslett chose to highlight ‘the extra cost of a caesarean…or its carbon footprint’ as an unacceptable reason for prioritising these factors—fiscal or environmental—in decision‐making above ‘a woman's right to choose her own medical care’ (Cosslett 2024). The fact that this environmental concern is raised in the context of an otherwise unrelated issue (the Green Party policy made no reference to the carbon footprint of maternity care), speaks to a wider public anxiety/suspicion that environmental considerations may be used opportunistically to drive agendas fulfilling other priorities (Hall 2024)—something we observed in our focus groups. In fact, this speaks to a wider challenge: that issues of public distrust in health and environmental public policies may be much more intractable than any specific co‐benefits framing will be able to address. Broader issues of public trust in the UK healthcare system and trust in health policy more broadly is at a historic low (Gardner 2024). Although co‐benefits (or indeed co‐harm) approaches may be used to promote or explain policy measures, careful attention will need to be paid to how co‐benefits framings are mobilised, and by whom, if the aim is to ensure public support and trust for such initiatives.

The subjective nature of co‐benefits approaches, alongside issues of (dis)trust, raises a key policy‐related question: who should determine which health and care needs—needs that our findings and others have shown to reflect differing preferences among patients (Tronto 2010)—should align with co‐benefits policies associated with mitigating the environmental harms of healthcare? Our participants richly grappled with the complexities of this question. They were able to quite sophisticatedly reflect on the various challenges of adopting a co‐benefits approach, including issues of subjectivities, as well as the fact that such a framing often failed to provide solutions to problems because priorities still came into tension with one another. (The persistence of conflicts recognised by our participants has also been seen elsewhere (Redvers et al. 2024), including in the broader planetary and one health literature^16^, and has led to some in other sectors abandoning this policy framing completely, especially because it does not resolve tensions when problems become increasingly complex (Hegwood et al. 2022).)

Participants' appetite and capability to grapple with the challenges associated with co‐benefits framings suggest value in including public and patient voices in policymaking decisions about such approaches, and more broadly, with policies designed to address the environmental harms associated with healthcare. Such an approach to policymaking would allow a reflexive discussion on the range of subjectivities and complexities inherent in any co‐benefits approach adopted. Our focus group findings suggest that members of the public and patients would be particularly adept to such discussions as participants were able to develop their thinking together, and this helped to reveal and sometimes challenge subjective views. In fact, the perceptiveness and careful consideration that participants brought to the exercise could be a key attribute in developing and robustly improving any co‐benefits framework.

Such public and patient insights could then be integrated with the work of various scholars who have critically engaged with co‐benefits framings to suggest ways in which they can be legitimately acknowledged as a useful way to conceptualise policy directives. Kovacic et al. (2019), for example, argue that a co‐benefits framing must be seen as a policy *process* rather than a solution; ‘as experiments that may go wrong and may need to be corrected’ (p176)). Complexity and uncertainty, they say, can be paralysing because every policy action may have unintended consequences, trade‐offs and/or other long‐term aspects that are yet unknowns when they enter a complicated and uncertain socio‐political context within which they aim to be delivered. Therefore, they suggest a deliberative policy *process*, which should involve the continued reflection and evaluation of any co‐benefits policies. We argue that such a process could include public and patient involvement. Any unintended impacts could be addressed through ‘flanking policies’ to correct/re‐balance policy outcomes more favourably (Hildingsson and Johansson 2016). Another approach, proposed by Ürge‐Vorsatz et al. (2014), is to shift the language from ‘co‐benefits’ to ‘co‐impacts’ (p304). The term ‘impacts’, they argue, does not assume overall positive outcomes, acknowledging that policies typically have multiple intended and unintended outcomes, and encouraging wider consideration of such policy impacts. Incorporating public and patient views and insights on the use of this (and other) terms, and in particular, whether such linguistic changes could/would address any of the challenges associated with the co‐benefits approach, could also be an invaluable aspect of any political decision‐making process.

## 4 Conclusion

In developing co‐benefits policy approaches in healthcare, policymakers need to consider the subjectivity and context‐specificity of the way in which co‐benefits are constructed and understood. To do so, it is crucial to include multiple perspectives (including members of the public and patients) when developing co‐benefits policies, so as to better understand the various subjectivities and contexts associated with a particular co‐benefits framing, and to address the pervasive public mistrust in both climate and health policy making. Once a framing is chosen, decisions and their underlying assumptions—including how tensions were resolved—must be made open and transparent. This is not least because tensions are always a necessary or even essential characteristic of social and moral deliberation (Williams 1981) and will often persist where pluralistic values converge on an issue; the challenge for policymakers will be to determine how best to minimise such tensions.

## Author Contributions

**Gabrielle Samuel:** conceptualization (equal), data curation (equal), formal analysis (equal), funding acquisition (equal), methodology (equal), project administration (equal), writing – original draft (equal), writing – review and editing (equal). **Miranda MacFarlane:** formal analysis (equal), investigation (equal), methodology (equal), project administration (equal), writing – original draft (equal). **Sarah Briggs:** conceptualization (equal), data curation (equal), formal analysis (equal), funding acquisition (equal), methodology (equal), writing – review and editing (equal).

## Ethics Statement

Approval was granted by King's College Research Ethics Committee (LRS/DP‐23.24–39889).

## Consent

Informed consent was obtained from all individual participants included in the study. The authors affirm that participants provided informed consent for publication.

## Conflicts of Interest

The authors declare no conflicts of interest .

## Data Availability

The datasets used and/or analysed during the current study are available from the corresponding author on reasonable request and in line with consent requirements.

## Endnotes

^1^

https://practicegreenhealth.org/.

^2^

https://www.todaesolar.com.au/blacktown‐hospital‐760kw‐rooftop‐installation/.

^3^
Including by ‘assessing health systems’ GHG emissions, and developing action plans, nationally determined decarbonisation targets, and procurement standards for national health systems, including supply chains'.

^4^
NHS England's Net Zero strategy aims to reach net zero emissions across sources directly and indirectly controlled by the NHS in 2040, and for emissions which the NHS can influence, to achieve net zero by 2045. See https://www.england.nhs.uk/greenernhs/a‐net‐zero‐nhs/. NHS Scotland brought forward an earlier target for reaching net zero emissions across the NHS from 2045 to 2040. See https://www.gov.scot/binaries/content/documents/govscot/publications/strategy‐plan/2022/08/nhs‐scotland‐climate‐emergency‐sustainability‐strategy‐2022‐2026/documents/nhs‐scotland‐climate‐emergency‐sustainability‐strategy‐2022‐2026/nhs‐scotland‐climate‐emergency‐sustainability‐strategy‐2022‐2026/govscot%3Adocument/nhs‐scotland‐climate‐emergency‐sustainability‐strategy‐2022‐2026.pdf. NHS Wales has a target to reach net zero by 2030, falling under the target for all Welsh public sector organisations. Nationally Wales has a target to reach net zero across all sectors by 2050. See https://www.gov.wales/sites/default/files/publications/2021‐03/nhs‐wales‐decarbonisation‐strategic‐delivery‐plan.pdf. NHS Northern Ireland does not have a distinct net zero strategy, and falls under the targets outlined in the Northern Ireland Climate Change Act (2022) for all sectors to reach net zero by 2050. For information on the Climate Change Act, see: https://www.daera‐ni.gov.uk/articles/climate‐change‐act‐northern‐ireland‐2022‐key‐elements.

^5^

https://www.legislation.gov.uk/ukpga/2008/27/contents.

^6^

https://www.legislation.gov.uk/ukpga/2022/31/contents.

^7^
See https://www.england.nhs.uk/greenernhs/get‐involved/suppliers/#:~:text=Net%20zero%20supplier%20roadmap&text=From%20April%202023%3A%20for%20all,with%20PPN%2006%2F21.

^8^

https://www.legislation.gov.uk/asp/2009/12/contents.

^9^

https://www.legislation.gov.uk/asp/2019/15/contents/enacted.

^10^

https://www.legislation.gov.uk/asp/2024/15/enacted.

^11^

https://www.futuregenerations.wales/about‐us/future‐generations‐act/.

^12^

https://www.gov.wales/section‐6‐biodiversity‐and‐resilience‐ecosystems‐duty‐summary‐report‐2022‐html.

^13^

https://www.legislation.gov.uk/anaw/2015/2/contents/enacted.

^14^

https://www.the‐liminal‐space.com/all‐projects/netzeronhs.

^15^
Green Party policy prior to the publication of their 2024 election manifesto in July 2024 included a section on maternity services, including the statement ‘The incidence of medical intervention in childbirth has escalated in recent years, particularly the rate of caesarean sections, which are expensive and, when not medically required, risky. We will work to reduce the number of interventions in childbirth, and change the culture of the NHS so that birth is treated as a normal and non‐medical event, in which mothers are empowered and able to be in control’: https://x.com/JamesTitcombe/status/1797169491048026447.

^16^
Planetary and one health both broadly refer to the idea that to improve human health, humans' interconnections with animals, plants, and the environment must also be recognised. The health of humans, non‐humans and the planet are interrelated.

## References

[shil70058-bib-0001] Alliance for Transformative Action on Climate and Health . n.d. https://www.who.int/initiatives/alliance‐for‐transformative‐action‐on‐climate‐and‐health/commitments.

[shil70058-bib-0002] Alshqaqeeq, F. , C. Mcguire , M. Overcash , K. Ali , and J. Twomey . 2020. “Choosing Radiology Imaging Modalities to Meet Patient Needs With Lower Environmental Impact.” Resources, Conservation and Recycling 155: 104657. 10.1016/j.resconrec.2019.104657.

[shil70058-bib-0003] Amelung, D. , H. Fischer , A. Herrmann , et al. 2019. “Human Health as a Motivator for Climate Change Mitigation: Results From Four European High‐Income Countries.” Global Environmental Change 57: 101918. 10.1016/j.gloenvcha.2019.05.002.

[shil70058-bib-0004] Andrieu, B. , L. Marrauld , O. Vidal , M. Egnell , L. Boyer , and G. Fond . 2023. “Health‐Care Systems' Resource Footprints and Their Access and Quality in 49 Regions Between 1995 and 2015: An Input‐Output Analysis.” Lancet Planetary Health 7, no. 9: e747–e758. 10.1016/s2542-5196(23)00169-9. 37673545 PMC10495829

[shil70058-bib-0005] Bacchi, C. 2009. Analysing Policy. Pearson Higher Education.

[shil70058-bib-0006] Bhopal, A. , K. Bærøe , and O. F. Norheim . 2025. “Ambition With Uncertainty: Exploring Policy‐Makers’ Perspectives on Pathways to Net Zero Healthcare.” International Journal of Health Policy and Management 14: 8440: Online first. 10.34172/ijhpm.8440. PMC11845860 40767189

[shil70058-bib-0007] Bullington, J. , and I. Fagerberg . 2013. “The Fuzzy Concept of ‘Holistic Care’: A Critical Examination.” Scandinavian Journal of Caring Sciences 27, no. 3: 493–494. 10.1111/scs.12053. 23844596

[shil70058-bib-0008] Burt, J. , and G. Cameron . 2024. The NHS Net Zero Target 4 Years on: Have Public Perceptions Changed?. Health Foundation. https://www.health.org.uk/features‐and‐opinion/blogs/the‐nhs‐net‐zero‐target‐4‐years‐on‐have‐public‐perceptions‐changed?utm_source=emai&utm_medium=email&utm_campaign=net_zero_roundup&dm_i=4Y2,8TI0E,9XMG5P,10OYNG,1.

[shil70058-bib-0009] Cáceres, D. M. , F. Silvetti , and S. Díaz . 2016. “The Rocky Path From Policy‐Relevant Science to Policy Implementation—A Case Study From the South American Chaco.” Current Opinion in Environmental Sustainability 19: 57–66. 10.1016/j.cosust.2015.12.003.

[shil70058-bib-0010] Callan, C. , T. Hardie , and G. Cameron . 2023. Net Zero NHS: What Does the Public Think?. Health Foundation. https://www.health.org.uk/features‐and‐opinion/blogs/net‐zero‐nhs‐what‐does‐the‐publicthink#:~:text=Support%20for%20the%20NHS%20net%20zero%20ambition%20has%20fallen%20to%2060%25&text=18%25%20are%20aware%20of%20this,and%20only%2012%25%20oppose%20it.

[shil70058-bib-0011] Callan, C. , T. Hardie , and G. Cameron . 2024. Policy Levers for a Net Zero NHS: Four Priorities for the Future. Health Foundation. https://www.health.org.uk/reports‐and‐analysis/briefings/policy‐levers‐for‐a‐net‐zero‐nhs‐four‐priorities‐for‐the‐future.

[shil70058-bib-0012] Carbon Trust . 2021. NHS Wales, Decarbonisation Strategic Delivery Plan. https://www.gov.wales/sites/default/files/publications/2021‐03/nhs‐wales‐decarbonisation‐strategic‐delivery‐plan.pdf.

[shil70058-bib-0014] COP28 . 2023. COP28 UAE Declaration on Climate and Health. https://www.cop28.com/en/cop28‐uae‐declaration‐on‐climate‐and‐health.

[shil70058-bib-0015] Cosslett, R. L. 2024. “How Did the Greens Get it So Wrong on ‘natural’ Birth?” Guardian. https://www.theguardian.com/commentisfree/article/2024/jun/07/women‐birth‐green‐party‐natural‐c‐sections‐too‐posh‐to‐push.

[shil70058-bib-0016] Coxon, K. , A. Chisholm , R. Malouf , R. Rowe , and J. Hollowell . 2017. “What Influences Birth Place Preferences, Choices and Decision‐Making Amongst Healthy Women With Straightforward Pregnancies in the UK? A Qualitative Evidence Synthesis Using a ‘Best Fit’ Framework Approach.” BMC Pregnancy and Childbirth 17, no. 1: 103. 10.1186/s12884-017-1279-7. 28359258 PMC5374625

[shil70058-bib-0017] Cussans, A. , Harvey, G. , Kemple, T. & Tomson, M. 2021. “Interventions to Reduce the Environmental Impact of Medicines: A UK Perspective.” Journal of Climate Change and Health, 4, 100079. 10.1016/j.joclim.2021.100079.

[shil70058-bib-0018] Davies, J. F. , F. Mcgain , E. Sloan , J. Francis , and S. Best . 2024. “A Qualitative Exploration of Barriers, Enablers, and Implementation Strategies to Replace Disposable Medical Devices With Reusable Alternatives.” Lancet Planetary Health 8, no. 11: e937–e945. 10.1016/s2542-5196(24)00241-9. 39515352

[shil70058-bib-0019] Dinh, N. T. T. , J. Tran , and M. Hensher . 2024. “Measuring and Valuing the Health Co‐Benefits of Climate Change Mitigation: A Scoping Review.” Lancet Planetary Health 8, no. 6: e402–e409. 10.1016/s2542-5196(24)00095-0. 38849182

[shil70058-bib-0020] Dubash, N. K. , D. Raghunandan , G. Sant , and A. Sreenivas . 2013. “Indian Climate Change Policy: Exploring a Co‐Benefits Based Approach.” Economic and Political Weekly: 47–61.

[shil70058-bib-0021] Gao, J. , S. Kovats , S. Vardoulakis , et al. 2018. “Public Health Co‐Benefits of Greenhouse Gas Emissions Reduction: A Systematic Review.” Science of the Total Environment 627: 388–402. 10.1016/j.scitotenv.2018.01.193. 29426161

[shil70058-bib-0022] Gardner, T. 2024. The Government Has Lost the Public’s Trust on the NHS – What Might Win it Back? Health Foundation. https://www.health.org.uk/features‐and‐opinion/blogs/the‐government‐has‐lost‐the‐public‐s‐trust‐on‐the‐nhs‐what‐might‐win‐it.

[shil70058-bib-0023] Garrett, S. M. , J. Hilder , R. Tester , et al. 2024. “Young People Talk About Digital Support for Mental Health: An Online Survey of 15–30‐Year Olds in New Zealand.” Health Expectations 27, no. 4: e70001. 10.1111/hex.70001. 39188108 PMC11347751

[shil70058-bib-0024] Gourevitch, R. A. , A. Mehrotra , G. Galvin , M. Karp , A. Plough , and N. T. Shah . 2017. “How Do Pregnant Women Use Quality Measures When Choosing Their Obstetric Provider?” Birth 44, no. 2: 120–127. 10.1111/birt.12273. 28124390 PMC5484308

[shil70058-bib-0025] Greenvironment 2021. Real Time Monitoring of Hospital Wastewater: A Good Deal to Nature and Saving Water Spend. https://greenvironmentindia.com/real‐time‐monitoring‐of‐hospital‐wastewater‐a‐good‐deal‐to‐nature‐and‐saving‐water‐spend/.

[shil70058-bib-0026] Haines, A. 2017. “Health Co‐Benefits of Climate Action.” Lancet Planetary Health 1: e4–e5. 10.1016/s2542-5196(17)30003-7. 29851591

[shil70058-bib-0027] Hall, A. G. 2024. “The Green Party’s Stance on C‐Sections Shows How Little Women’s Births Are Understood.” Grazia. https://graziadaily.co.uk/life/parenting/green‐party‐childbirth/.

[shil70058-bib-0028] Ham, C. 2023. The Rise and Decline of the NHS in England 20.0–20. King’s Fund. https://assets.kingsfund.org.uk/f/256914/x/0ab966500b/rise_decline_nhs_england_2000‐20_2023.pdf.

[shil70058-bib-0029] Health Care without Harm . 2021. “Global Road Map for Health Care Decarbonization, Annex C: Interventions.” Health Care without Harm. https://noharm‐global.org/articles/news/global/our‐new‐road‐map‐zero‐emissions‐health‐care.

[shil70058-bib-0030] Hegwood, M. , R. E. Langendorf , and M. G. Burgess . 2022. “Why Win–Wins Are Rare in Complex Environmental Management.” Nature Sustainability 5, no. 8: 674–680. 10.1038/s41893-022-00866-z.

[shil70058-bib-0031] Hildingsson, R. , and B. Johansson . 2016. “Governing Low‐Carbon Energy Transitions in Sustainable Ways: Potential Synergies and Conflicts Between Climate and Environmental Policy Objectives.” Energy Policy 88: 245–252. 10.1016/j.enpol.2015.10.029.

[shil70058-bib-0032] Holguera, J. G. , N. Niwa , and P. N. Senn . 2020. “Health & Environment Co‐Benefits: Concepts and Recommendations for Clinical Practice.” Revue Medicale Suisse 8: 5–35. 34164965

[shil70058-bib-0013] IPPC . 2014. Mitigation of Climate Change. Contribution of Working Group III to the Fifth Assessment Report of the Intergovernmental Panel on Climate Change, Vol. 1454, 147.

[shil70058-bib-0033] Issa, R. , C. Forbes , C. Baker , et al. 2024. “Sustainability Is Critical for Future Proofing the NHS.” BMJ 385: e079259. 10.1136/bmj-2024-079259. 38604667

[shil70058-bib-0034] Karliner, J. , S. Slotterback , R. Boyd , B. Ashby , K. Steele , and J. Wang . 2020. “Health Care’s Climate Footprint: The Health Sector Contribution and Opportunities for Action.” Supplement. European Journal of Public Health 30, no. S5: ckaa165.843. 10.1093/eurpub/ckaa165.843.

[shil70058-bib-0035] Karlsson, M. , E. Alfredsson , and N. Westling . 2020. “Climate Policy Co‐Benefits: A Review.” Climate Policy 20, no. 3: 292–316. 10.1080/14693062.2020.1724070.

[shil70058-bib-0036] King’s Green Plan . n.d. https://www.kch.nhs.uk/about/our‐strategy/kings‐green‐plan/.

[shil70058-bib-0037] Kovacic, Z. , R. Strand , and T. Voöker . 2019. The Circular Economy in Europe: Critical Perspectives on Policies and Imaginaries. Routledge.

[shil70058-bib-0038] Krens, J. 2022. “New Dutch Green Deal Signed to Boost Sustainability in Healthcare.” Pinsent Masons. https://www.pinsentmasons.com/out‐law/news/new‐dutch‐green‐deal‐signed‐to‐boost‐sustainability‐in‐healthcare.

[shil70058-bib-0039] Lemery, J. , K. Knowlton , and C. Sorensen . 2021. “Chapter 19 Health Co‐Benefits of Climate Mitigation Strategies.” In Global Climate Change and Human Health: From Science to Practice. John Wiley & Sons: Incorporated.

[shil70058-bib-0040] Lupton, D. , and M. Michael . 2017. “Depends on Who’s Got the Data’: Public Understandings of Personal Digital Dataveillance.” Surveillance and Society 15, no. 2: 254–268. 10.24908/ss.v15i2.6332.

[shil70058-bib-0041] Maibach, E. W. , M. Nisbet , P. Baldwin , K. Akerlof , and G. Diao . 2010. “Reframing Climate Change as a Public Health Issue: An Exploratory Study of Public Reactions.” BMC Public Health 10, no. 1: 299. 10.1186/1471-2458-10-299. 20515503 PMC2898822

[shil70058-bib-0042] Marselle, M. R. , T. Hartig , D. T. C. Cox , et al. 2021. “Pathways Linking Biodiversity to Human Health: A Conceptual Framework.” Environment International 150: 106420. 10.1016/j.envint.2021.106420. 33556912

[shil70058-bib-0043] Marshall, L. , and A. Allen . 2023. Health and Climate Change: Complex Problems With Co‐Benefits. https://www.health.org.uk/publications/long‐reads/health‐and‐climate‐change‐complex‐problems‐with‐co‐benefits?gad_source=1&gclid=CjwKCAjwodC2BhAHEiwAE67hJLYfn7gB0NYvOqk3nF8O1RR0KfvDwUFlvmAjwGUVc‐cTEpX6VIusIxoCigsQAvD_BwE.

[shil70058-bib-0044] Mayrhofer, J. P. , and J. Gupta . 2016. “The Science and Politics of Co‐Benefits in Climate Policy.” Environmental Science & Policy 57: 22–30. 10.1016/j.envsci.2015.11.005.

[shil70058-bib-0045] Montgomery, A. , and T. Fahey . 2001. “How Do Patients' Treatment Preferences Compare With Those of Clinicians?” Supplement. BMJ Quality and Safety 10, no. S1: i39–i43. 10.1136/qhc.0100039. PMC1765739 11533437

[shil70058-bib-0046] Moura‐Neto, J. A. , K. Barraclough , and J. W. M. Agar . 2019. “A Call‐To‐Action for Sustainability in Dialysis in Brazil.” Jornal Brasileiro de Nefrologia 41, no. 4: 560–563. 10.1590/2175-8239-jbn-2019-0014. 31268113 PMC6979574

[shil70058-bib-0047] Moutet, L. , P. Bernard , R. Green , et al. 2024. “The Public Health Co‐Benefits of Strategies Consistent With Net‐Zero Emissions: A Systematic Review of Quantitative Studies.” medRxiv: 2024.08.26.24312597. 10.1016/S2542-5196(24)00330-9 PMC7618420 39954682

[shil70058-bib-0048] Myers, T. A. , M. C. Nisbet , E. W. Maibach , and A. A. Leiserowitz . 2012. “A Public Health Frame Arouses Hopeful Emotions About Climate Change.” Climatic Change 113, no. 3–4: 1105–1112. 10.1007/s10584-012-0513-6.

[shil70058-bib-0049] Namburar, S. , M. Pillai , G. Varghese , C. Thiel , and A. L. Robin . 2018. “Waste Generated During Glaucoma Surgery: A Comparison of Two Global Facilities.” American Journal of Ophthalmology Case Reports 12: 87–90. 10.1016/j.ajoc.2018.10.002. 30364583 PMC6197147

[shil70058-bib-0050] National Institute for Health and Care Excellence (Nice) . 2023. NICE Listens: Public Consultation on Environmental Sustainability. https://www.nice.org.uk/Media/Default/Get‐involved/NICE‐listens/Environmental%20sustainability%20final%20report.docx. 39190730

[shil70058-bib-0051] NHS . 2020. “Delivering a ‘Net Zero’.” National Health Service. https://www.england.nhs.uk/greenernhs/wp‐content/uploads/sites/51/2020/10/delivering‐a‐net‐zero‐national‐health‐service.pdf.

[shil70058-bib-0052] NHS England 2018. Reducing the Use of Natural Resources in Health and Social Care. Sustainable Development Unit. https://networks.sustainablehealthcare.org.uk/sites/default/files/resources/20180912_Health_and_Social_Care_NRF_web.pdf.

[shil70058-bib-0053] NHS England . 2023. Net Zero Travel and Transport Strategy. https://www.england.nhs.uk/long‐read/net‐zero‐travel‐and‐transport‐strategy/.

[shil70058-bib-0054] NHS Scotland (2022). Climate Emergency & Sustainability Strategy. https://www.gov.scot/binaries/content/documents/govscot/publications/strategy‐plan/2022/08/nhs‐scotland‐climate‐emergency‐sustainability‐strategy‐2022‐2026/documents/nhs‐scotland‐climate‐emergency‐sustainability‐strategy‐2022‐2026/nhs‐scotland‐climate‐emergency‐sustainability‐strategy‐2022‐2026/govscot%3Adocument/nhs‐scotland‐climate‐emergency‐sustainability‐strategy‐2022‐2026.pdf.

[shil70058-bib-0055] Oliveira, R. V. , and M. Thorseth . 2016. Ethical Implications of Co‐benefits Rationale within Climate Change Mitigation Strategy, 141–170. Nordic Journal of Applied Ethics.

[shil70058-bib-0056] Pan American Health Organisation . n.d. Climate Change and Health. https://www.paho.org/en/topics/climate‐change‐and‐health.

[shil70058-bib-0057] Phelan, J. C. , B. G. Link , and P. Tehranifar . 2010. “Social Conditions as Fundamental Causes of Health Inequalities: Theory, Evidence, and Policy Implications.” Supplement. Journal of Health and Social Behavior 51, no. Sl: S28–S40. 10.1177/0022146510383498. 20943581

[shil70058-bib-0058] Public Health Wales . 2024. Our Decarbonisation and Sustainability Plan 20.4–2026. https://phw.nhs.wales/about‐us/working‐together‐for‐a‐healthier‐wales/our‐decarbonisation‐and‐sustainability‐plan‐2024‐2026/.

[shil70058-bib-0059] Ravindrane, R. , and J. Patel . 2022. “The Environmental Impacts of Telemedicine in Place of Face‐To‐Face Patient Care: A Systematic Review.” Future Healthcare Journal 9, no. 1: 28–33. 10.7861/fhj.2021-0148. PMC8966787 35372776

[shil70058-bib-0060] Rayner, T. , E. Smith , C. Howarth , and J. Graham . 2025. Advancing and Integrating Climate and Health Policies in the United Kingdom: Insights from National Stakeholders. University of East Anglia and Grantham Research Institute on Climate Change and the Environment. 10.17605/OSF.IO/SQ3R7.

[shil70058-bib-0061] Redvers, N. 2021. “Patient‐Planetary Health Co‐Benefit Prescribing: Emerging Considerations for Health Policy and Health Professional Practice.” Frontiers in Public Health 9: 678545. 10.3389/fpubh.2021.678545. 33996734 PMC8119779

[shil70058-bib-0062] Redvers, N. , J. Hartmann‐Boyce , and S. Tonkin‐Crine . 2024. “Patient‐Planetary Health Co‐Benefit Prescribing in a Circumpolar Health Region: A Qualitative Study of Physician Voices From the Northwest Territories, Canada.” BMJ Open 14, no. 3: e081156. 10.1136/bmjopen-2023-081156. PMC10910660 38431297

[shil70058-bib-0063] Resnik, D. B. 2015. “Bioethical Issues in Providing Financial Incentives to Research Participants.” Medicolegal and Bioethics 5: 35–41. 10.2147/mb.s70416. 26807399 PMC4719771

[shil70058-bib-0064] Robinson, J. M. , and M. F. Breed . 2019. “Green Prescriptions and Their Co‐Benefits: Integrative Strategies for Public and Environmental Health.” Challenges (Middletown, Conn.) 10, no. 1: 9. 10.3390/challe10010009.

[shil70058-bib-0065] Rose, G. 2008. Rose’s Strategy of Preventive Medicine. Oxford University Press.

[shil70058-bib-0066] Royal College of Physicians . 2024. Green Physician Toolkit. https://www.rcp.ac.uk/media/0lppfhmw/rcp‐green‐physician‐toolkit.pdf.

[shil70058-bib-0067] Ryan, S. M. , and C. J. Nielsen . 2010. “Global Warming Potential of Inhaled Anesthetics: Application to Clinical Use.” Anesthesia & Analgesia 111, no. 1: 92–98. 10.1213/ane.0b013e3181e058d7. 20519425

[shil70058-bib-0068] Samuel, G. 2023. “UK Health Researchers’ Considerations of the Environmental Impacts of Their Data‐Intensive Practices and its Relevance to Health Inequities.” BMC Medical Ethics 24, no. 1: 90. 10.1186/s12910-023-00973-2. 37891541 PMC10612270

[shil70058-bib-0069] Samuel, G. , G. M. Anderson , F. Lucivero , and A. Lucassen . 2024. “Why Digital Innovation May Not Reduce Healthcare’s Environmental Footprint.” BMJ 385: e078303. 10.1136/bmj-2023-078303. 38830688 PMC7616622

[shil70058-bib-0070] Shrestha, P. , S. K. Nukala , F. Islam , T. Badgery‐Parker , and F. Foo . 2024. “The Co‐Benefits of Climate Change Mitigation Strategies on Cardiovascular Health: A Systematic Review.” Lancet Regional Health—Western Pacific 48: 101098. 10.1016/j.lanwpc.2024.101098. 39380746 PMC11458989

[shil70058-bib-0071] Sonnino, R. , and S. Mcwilliam . 2011. “Food Waste, Catering Practices and Public Procurement: A Case Study of Hospital Food Systems in Wales.” Food Policy 36, no. 6: 823–829. 10.1016/j.foodpol.2011.09.003.

[shil70058-bib-0072] Strain, D. 2022. “When the Gloves Come off.” British Medical Association. https://www.bma.org.uk/news‐and‐opinion/when‐the‐gloves‐come‐off.

[shil70058-bib-0073] Talbot, B. , R. A. Fletcher , B. Neal , et al. 2024. “The Potential for Reducing Greenhouse Gas Emissions Through Disease Prevention: A Secondary Analysis of Data From the CREDENCE Trial.” Lancet Planetary Health 8, no. 12: e1055–e1064. 10.1016/s2542-5196(24)00281-x. 39674195

[shil70058-bib-0074] Tennison, I. , S. Roschnik , B. Ashby , et al. 2021. “Health Care's Response to Climate Change: A Carbon Footprint Assessment of the NHS in England.” Lancet Planetary Health 5, no. 2: e84–e92. 10.1016/s2542-5196(20)30271-0. 33581070 PMC7887664

[shil70058-bib-0075] The Academy of Medical Sciences 2021. A Healthy Future—Tackling Climate Change Mitigation and Human Health Together. Academy of Medical Sciences. https://royalsociety.org/‐/media/policy/projects/climate‐change‐mitigation‐human‐health/ams‐climate‐change‐report.pdf.

[shil70058-bib-0076] The World Bank . n.d. Health and Climate Change. https://www.worldbank.org/en/topic/health/brief/health‐and‐climate‐change.

[shil70058-bib-0077] Thiel, C. L. , E. Schehlein , T. Ravilla , et al. 2017. “Cataract Surgery and Environmental Sustainability: Waste and Lifecycle Assessment of Phacoemulsification at a Private Healthcare Facility.” Journal of Cataract & Refractive Surgery 43, no. 11: 1391–1398. 10.1016/j.jcrs.2017.08.017. 29223227 PMC5728421

[shil70058-bib-0078] Thomas, F. , and M. Depledge . 2015. “Medicine ‘Misuse’: Implications for Health and Environmental Sustainability.” Social Science & Medicine 143: 81–87. 10.1016/j.socscimed.2015.08.028. 26344126

[shil70058-bib-0079] Tronto, J. 2010. “Creating Caring Institutions: Politics, Plurality, and Purpose.” Ethics and Social Welfare 4, no. 2: 158–171. 10.1080/17496535.2010.484259.

[shil70058-bib-0080] Ürge‐Vorsatz, D. , S. T. Herrero , N. K. Dubash , and F. Lecocq . 2014. “Measuring the Co‐Benefits of Climate Change Mitigation.” Annual Review of Environment and Resources 39, no. 1: 549–582. 10.1146/annurev-environ-031312-125456.

[shil70058-bib-0081] Vestesson, E. , K. De Corte , P. Chappell , E. Crellin , and G. M. Clarke . 2023. “Antibiotic Prescribing in Remote Versus Face‐To‐Face Consultations for Acute Respiratory Infections in Primary Care in England: An Observational Study Using Target Maximum Likelihood Estimation.” eClinicalMedicine 64: 102245. 10.1016/j.eclinm.2023.102245. 37842171 PMC10568332

[shil70058-bib-0082] Widdicks, K. , F. Lucivero , G. Samuel , et al. 2023. “Systems Thinking and Efficiency Under Emissions Constraints: Addressing Rebound Effects in Digital Innovation and Policy.” Patterns 4, no. 2: 100679. 10.1016/j.patter.2023.100679. 36873905 PMC9982294

[shil70058-bib-0083] Williams, B. 1981. Moral Luck: Philosophical Papers 1973‐1980. Cambridge University Press.

[shil70058-bib-0084] Workman, A. , G. Blashki , K. J. Bowen , D. J. Karoly , and J. Wiseman . 2018. “The Political Economy of Health Co‐Benefits: Embedding Health in the Climate Change Agenda.” International Journal of Environmental Research and Public Health 15, no. 4: 674: [Online]. 10.3390/ijerph15040674. 29617317 PMC5923716

[shil70058-bib-0085] World Health Organisation . 2023. Operational Framework for Building Climate Resilient and Low Carbon Health Systems. https://www.who.int/publications/i/item/9789240081888.

[shil70058-bib-0086] World Health Organisation . n.d. Health Co‐Benefits of Climate Action. https://www.who.int/teams/environment‐climate‐change‐and‐health/climate‐change‐and‐health/capacity‐building/toolkit‐on‐climate‐change‐and‐health/cobenefits.

[shil70058-bib-0087] Zikhathile, T. , H. Atagana , J. Bwapwa , and D. Sawtell . 2022. “A Review of the Impact That Healthcare Risk Waste Treatment Technologies Have on the Environment.” International Journal of Environmental Research and Public Health 19: 11967. 10.3390/ijerph191911967. 36231269 PMC9565833

[shil70058-bib-0088] Zimmermann, B. M. , H. Wagenaar , K. Kieslich , et al. 2022. “Democratic Research: Setting up a Research Commons for a Qualitative, Comparative, Longitudinal Interview Study During the COVID‐19 Pandemic.” SSM‐Qualitative Research in Health 2: 100158. 10.1016/j.ssmqr.2022.100158. 36092769 PMC9448682

